# CNPY2 inhibits MYLIP-mediated AR protein degradation in prostate cancer cells

**DOI:** 10.18632/oncotarget.24824

**Published:** 2018-04-03

**Authors:** Saya Ito, Akihisa Ueno, Takashi Ueda, Hideo Nakagawa, Hidefumi Taniguchi, Naruhiro Kayukawa, Atsuko Fujihara-Iwata, Fumiya Hongo, Koji Okihara, Osamu Ukimura

**Affiliations:** ^1^ Department of Urology, Graduate School of Medical Science, Kyoto Prefectural University of Medicine, Kyoto-City, Kyoto 602-8566, Japan; ^2^ Department of Urology, Uji Takeda Hospital, Uji-City, Kyoto 611-0021, Japan

**Keywords:** CNPY2, prostate cancer, AR, MYLIP, protein degradation

## Abstract

The androgen receptor (AR) is a ligand-dependent transcription factor that promotes prostate cancer (PC) cell growth through control of target gene expression. This report suggests that Canopy FGF signaling regulator 2 (CNPY2) controls AR protein levels in PC cells. We found that AR was ubiquitinated by an E3 ubiquitin ligase, myosin regulatory light chain interacting protein (MYLIP) and then degraded through the ubiquitin-proteasome pathway. CNPY2 decreased the ubiquitination activity of MYLIP by inhibition of interaction between MYLIP and UBE2D1, an E2 ubiquitin ligase. CNPY2 up-regulated gene expression of AR target genes such as *KLK3* gene which encodes the prostate specific antigen (PSA) and promoted cell growth of PC cells. The cell growth inhibition by CNPY2 knockdown was rescued by *AR* overexpression. Furthermore, positive correlation of expression levels between CNPY2 and AR/AR target genes was observed in tissue samples from human prostate cancer patients. Together, these results suggested that CNPY2 promoted cell growth of PC cells by inhibition of AR protein degradation through MYLIP-mediated AR ubiquitination.

## INTRODUCTION

Signaling by the androgen-induced androgen receptor (AR) promotes cell growth of prostate cancer (PC) cells. AR is a ligand-dependent transcription factor. Androgen-bound ARs exerts both genomic action and non-genomic action in PC cells. In genomic pathway, AR transported into the cellular nucleus, bind to chromatin and control the expression of target genes [1]. Alternatively, in non-genomic pathway, AR activate MAP kinase signaling pathway [2] and Src signaling pathway [3].

AR protein levels are controlled by both transcriptional regulation [4, 5] and post-transcriptional regulation [6]. Several reports showed that AR was marked with ubiquitins and degraded by the ubiquitin/proteasome system [6]. Protein degradation by the ubiquitin/proteasome system requires 3 types of enzymes and proceeds through 3 steps. First, ubiquitins are activated by E1 activating enzymes, then, ubiquitins are transferred to E2 conjugating enzymes from E1 enzymes and finally, E3 ligases function as substrate recognition modules of the system and are capable of interaction with both E2 enzymes and substrate [7]. Three AR ubiquitination sites have been reported. K845 and K847 are in the AR C-terminal region (AF-2) [8], whereas K311 is in the AR N-terminal region (AF-1) [9]. K845 and K847 sites are ubiquitinated by several E3 ligases such as RNF6, Siah2, SKP2, CHIP and MDM2 [8]. Ubiquitination of the K845 and K847 by SKP2, CHIP and MDM2 induce ubiquitin-mediated AR protein degradation, while RNF6 and Siah2-mediated ubiquitination of the K845 and K857 enhance AR transcriptional activity [8]. The K311 site was recently reported to be ubiquitinated by SKP2 [9]. Ubiquitination of the K311 is critical for both AR protein stability and AR transcriptional activity [9]. The E3 ligases that specifically recognize AR are attracting attention as therapeutic targets for treating PC [6]. Identification of novel E3 ubiquitin ligases may provide additional therapeutic targets for PC.

Previously, we screened a *Drosophila* PC model to identify novel regulators of PC cell growth and invasion [10]. We found that Canopy 2 (CNPY2) markedly promoted cell growth and invasion of PC cells [10]. CNPY2 is a member of the Canopy family (which includes CNPY1, 2, 3 and 4) that contains a four amino acid sequence at the C-terminus that resembles the classical KDEL motif for endoplasmic reticulum (ER) retention [11]. Although the biological function of CNPY2 is unclear, CNPY2 is also called MIR [myosin regulatory light chain (MRLC) interacting protein]-interacting saposin-like protein (MSAP) based on findings that MSAP interacts with MIR *in vitro* [11]. MIR is also known as MYLIP and contains a RING domain in its C-terminal region [12]. MYLIP functions as an E3 ubiquitin ligase and promotes degradation of MRLC and low-density lipoprotein receptor (LDLR) proteins through the ubiquitin/proteasome pathway [12, 13]. MYLIP interacts with the UBE2D family of E2 ubiquitin conjugating enzymes (UBE2D1-4) through its RING domain and promotes ubiquitination of its substrate proteins [14, 15]. The finding that CNPY2 overexpression increased protein levels of MRLC and LDLR suggested that CNPY2 can regulate the protein stability by preventing MYLIP-mediated ubiquitination [11, 16]. However, the mechanism by which CNPY2 inhibits E3 ligase activity of MYLIP is unknown.

We found that MYLIP is a novel E3 ubiquitin ligase that recognizes and ubiquitinates AR. The MYLIP-mediated AR ubiquitination induced AR protein degradation by ubiquitin/proteasome system. We also showed that CNPY2 repressed the MYLIP-mediated AR ubiquitination through inhibiting the interaction between E2 and E3 ubiquitin enzymes. Thus, our study suggested that CNPY2 promoted cell growth of PC cells through regulating AR protein degradation via inhibition of ubiquitin/proteasome pathway.

## RESULTS

### AR protein level is decreased by CNPY2 knockdown in PC cells

**Figure 1:**
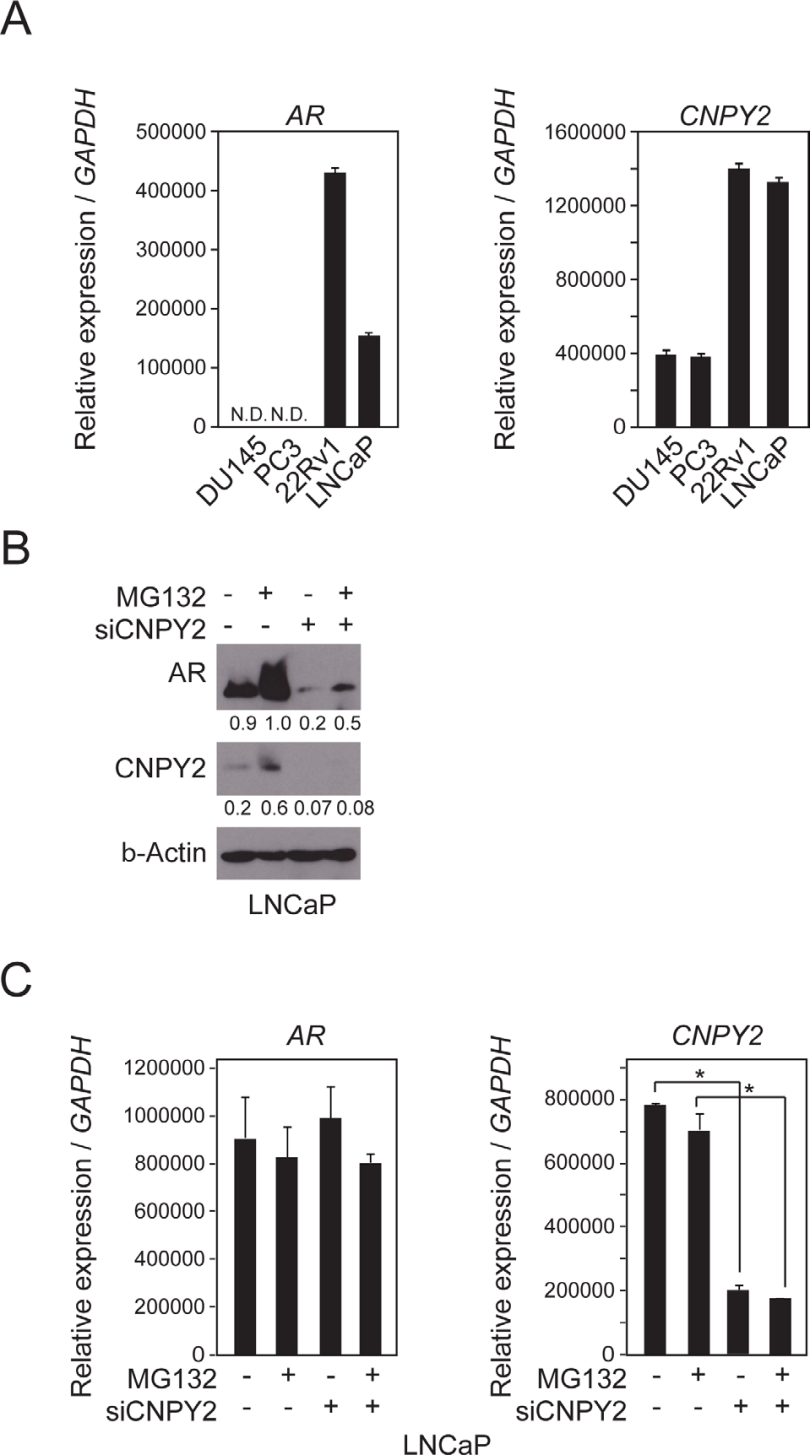
AR protein level is reduced by CNPY2 knockdown in PC cells **(A)** Expression of *CNPY2* and *AR* mRNAs in PC cells was measured by qPCR. Each measurement shows the average values of 3 independent measurements that were normalized to *GAPDH* mRNA expression levels. N.D., not detected. **(B)** Immunoblots of lysates from *CNPY2*-knockdown LNCaP cells cultured with 10 µM MG132 for 4 h. Band intensity was quantified by Adobe Photoshop. The measurements were normalized to each of β-Actin levels that are indicated at the bottom of each band. **(C)** mRNA expression of *CNPY2*-knockdown LNCaP cells cultured with 10 µM MG132 for 4 h prior to cell extraction. ^*^P < 0.05.

To investigate CNPY2 function, we used human prostate cancer cell lines to model the disease. The expression levels of the *CNPY2* transcript in the cell lines were first quantified by RT-PCR (Figure 1A). DU145, PC3 and LNCaP cell lines originated from prostate cancer cells that had metastasized to various tissues [17–19]. The 22Rv1 cell line originated from a primary prostate cancer [20]. The expression levels of *CNPY2* transcripts were higher in 22Rv1 and LNCaP than the other cell lines. Previous reports and our data showed that both 22Rv1 and LNCaP cells express AR transcripts [21], whereas DU145 and PC3 do not [22] (Figure 1A). Based on the *CNPY2* and *AR* expression signature in the different cell lines, we hypothesized that AR expression was regulated by *CNPY2* expression in prostate cancer. To assess whether AR expression was regulated by CNPY2, immunoblotting was performed using CNPY2 knockdown cells with an anti-AR antibody (Figure 1B). As the results, AR protein level was reduced by CNPY2 knockdown in LNCaP cells (Figure 1B) and 22Rv1 cells (data not shown). The decrease of AR protein level by CNPY2 knockdown was abrogated by treatment of the ubiquitin/proteasome inhibitor MG132 (Figure 1B). On the other hand, *AR* transcripts were not decreased by CNPY2 knockdown in LNCaP cells (Figure 1C) and 22Rv1 cells (data not shown). These results suggested that CNPY2 could inhibit AR protein degradation by the ubiquitin/proteasome system.

### CNPY2 represses MYLIP-mediated ubiquitination of AR

**Figure 2:**
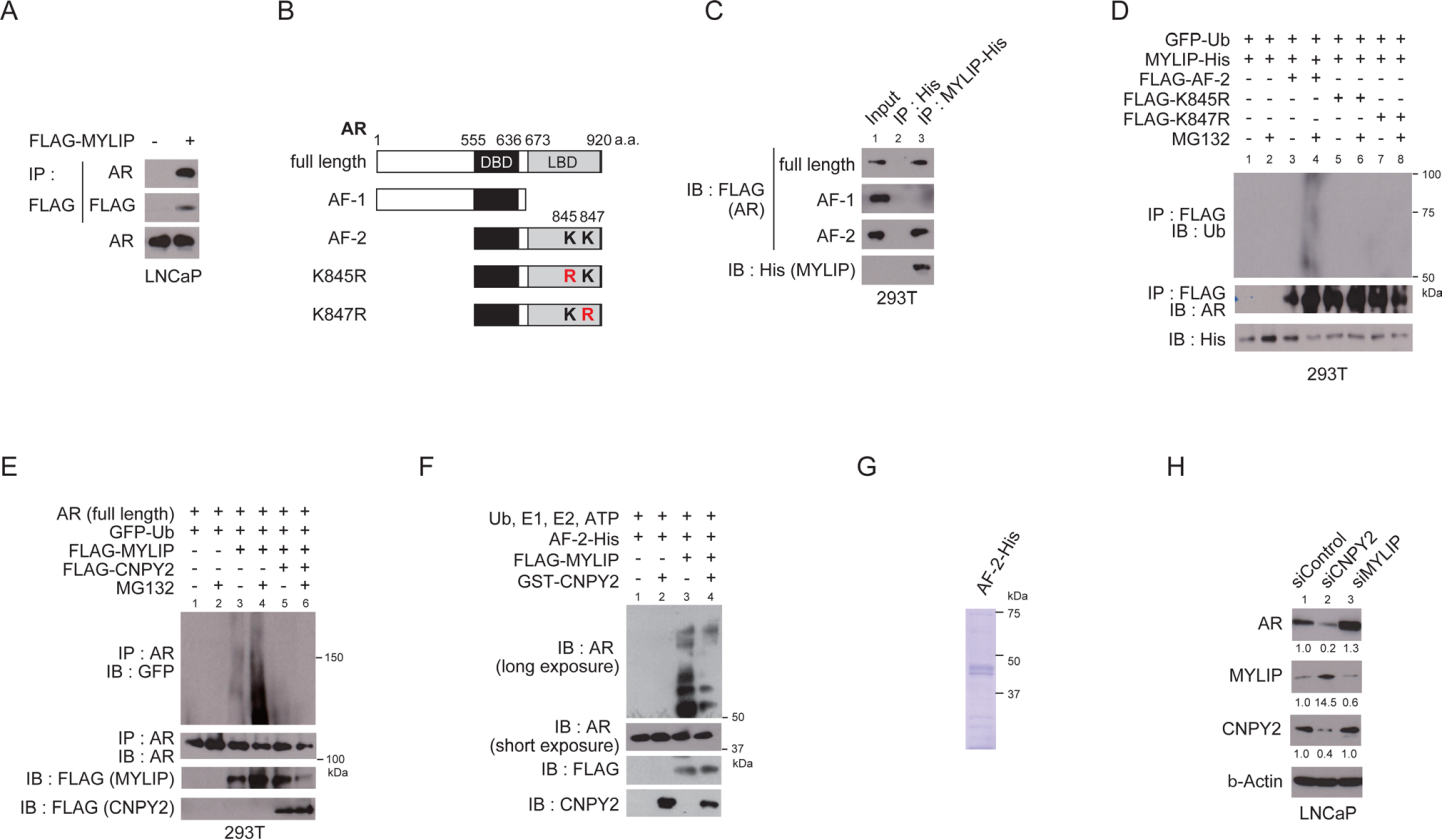
CNPY2 inhibits MYLIP-mediated AR ubiquitination and protein degradation **(A)** Immunoprecipitation of LNCaP cell extracts with anti-FLAG M2 affinity gel. LNCaP cells were transfected with FLAG-tagged MYLIP expression plasmids for 24 h and used for the immunoprecipitation. AR bound to MYLIP was then detected by immunoblotting. **(B)** Diagrams representing AR protein structure. K845 (Lys 845) and K847 (Lys847) are the two conserved ubiquitination sites on AR. DBD, DNA binding domain. LBD, Ligand binding domain. **(C)** Immunoprecipitation of 293T cell extracts with anti-His tag affinity beads. 293T cells were transfected with FLAG-tagged AR (full length, AF-1 or AF-2) expression plasmids and MYLIP-His or His-tag expression plasmids for 24 h and used for the immunoprecipitation. FLAG-ARs bound to MYLIP-His were then detected by immunoblotting with anti-FLAG. **(D)** MYLIP mediated-ubiquitination of AR was detected by *in vivo* ubiquitination assay. 293T cells were transfected with FLAG-AR (AF-2, K845R or K847R), MYLIP-His and EGFP-ubiquitin expression plasmids for 24 h and 10 µM MG132 was added to the culture medium 5 h before cell extraction. Cells were lysed and subjected to immunoprecipitation using anti-FLAG M2 affinity gel, followed by immunoblotting with each antibody. **(E)** *In vivo* ubiquitination assays were performed using 293T cells transfected with plasmids as indicated. Immunoprecipitation of AR (full length) was done using anti-AR (N-20). **(F)** *In vitro* ubiquitination assays were performed using recombinant AR (AF-2)-His, recombinant GST-CNPY2 and immunoprecipitated with FLAG-MYLIP. Reactions were performed with recombinant E1 enzyme, E2 enzyme and ubiquitin at 37° C for 2 h. Ubiquitination of AR was detected by immunoblotting with anti-AR (C-19). **(G)** Coomassie Brilliant Blue staining with recombinant AR (AF-2)-His protein. **(H)** Immunoblots using CNPY2 or MYLIP-knockdown LNCaP cell lysates with anti-AR, anti-MYLIP, or anti-CNPY2 antibodies. Band intensity was quantified by Adobe Photoshop. The measurements were normalized to si-Control protein levels that are indicated at the bottom of each band.

CNPY2 was previously reported to decrease MYLIP protein levels and inhibit MYLIP’s ubiquitin E3 ligase activity [11, 16]. The MRLC or LDLR were shown to be a MYLIP target substrate [12, 13], although few other target substrates of MYLIP are known. Several E3 ligases are known to interact with AR [8], but whether MYLIP interacts with AR awaits investigation. We hypothesized that CNPY2 could inhibit MYLIP recognition of AR and in turn suppress AR degradation. To examine whether MYLIP interacts with AR in prostate cancer cells, we first performed immunoprecipitation assay using LNCaP cells transfected with flag-tagged MYLIP. The results of these assays showed that AR and MYLIP physically interacted in prostate cancer cells (Figure 2A). To determine interaction domain of AR with MYLIP, immunoprecipitaion was performed using 293T cells which co-expressed with His tag-fused MYLIP and each of FLAG tag-fused partial ARs (full length, AF-1 and AF-2; Figure 2B and 2C). As the results of immunoprecipitation using anti-His-tag beads, physical interaction of MYLIP with the C-terminal region of AR (AF-2) was consistently observed, while its association with the N-terminal region of AR (AF-1) was not detected (Figure 2B and 2C).

Next, to investigate whether MYLIP functions as an E3 ubiquitin ligase that recognizes the AR protein, *in vivo* ubiquitination assays were performed using 293T cells that do not express endogenous AR. Cell lysates of 293T cells transfected with plasmids expressing FLAG tag-fused AR (AF-2), MYLIP-His and EGFP-ubiquitin were extracted and immunoprecipitation using anti-FLAG was performed. Poly-ubiquitinated AR (AF-2) was increased in lysates from MYLIP-transfected cells that were treated with MG132 (Figure 2D). Lysine residues 845 and 847 in the AR C-terminal region are ubiquitination sites critical for AR transcriptional activation or ubiquitin-mediated degradation [8] . When either lysine 845 or 847 was replaced with arginine, neither AR mutant (K845R or K847R) served as a MYLIP substrate *in vivo* (Figure 2D).

**Figure 3:**
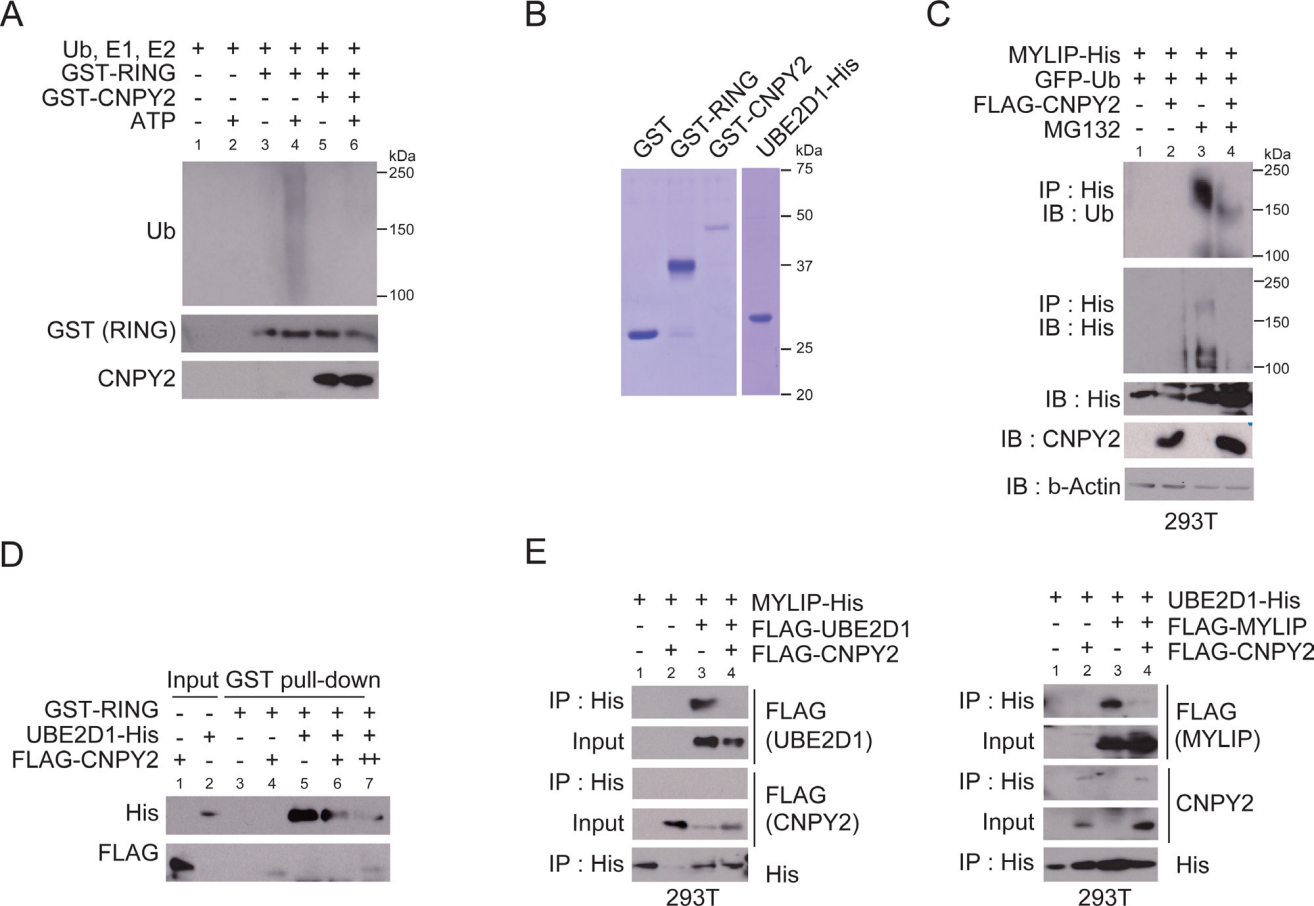
CNPY2 represses ubiquitination activity of MYLIP by inhibition of the interaction between MYLIP and UBE2D1 **(A)** *In vitro* ubiquitination assays were done with recombinant GST-RING protein and GST-CNPY2 protein. Ubiquitination was detected by immunoblotting using anti-ubiquitin antibody. **(B)** Coomassie Brilliant Blue staining with recombinant proteins for *in vitro* ubiquitination assay or GST pull-down assay. **(C)** *In vivo* ubiquitination assays were performed using 293T cells transfected with plasmids as indicated for 24 h. MG132 was added to the culture medium 5 h before cell extraction. Cells were lysed and subjected to immunoprecipitation using anti-His affinity beads, followed by immunoblotting with each antibody. **(D)** GST pull-down assays were performed with recombinant GST-RING and UBE2D1-His. Immunoprecipitated FLAG-CNPY2 was added to the reaction mixture. UBE2D1 bound to MYLIP was then detected by immunoblotting. **(E)** Immunoprecipitation was performed using 293T cells that were transfected with plasmids as indicated for 24 h. Immunoprecipitation using anti-His affinity beads, followed by immunoblotting with each antibody.

AR (full length) was also poly-ubiquitinated by MYLIP in an *in vivo* ubiquitination assay, and MYLIP-mediated AR poly-ubiquitination was inhibited by CNPY2 (Figure 2E). To determine whether CNPY2 could inhibit AR ubiquitination by MYLIP, *in vitro* ubiquitination assays were carried out with recombinant E1 enzyme, UBE2D1 (E2 enzyme), AR (AF-2) and immuprecipitated FLAG-MYLIP protein. We showed that AR (AF-2) was ubiquitinated by MYLIP protein *in vitro* (Figure 2F and 2G). Additionally, the MYLIP-mediated ubiquitination of AR was diminished by CNPY2 (Figure 2F and 3B).

Finally, to investigate whether CNPY2 inhibited MYLIP-mediated AR degradation in prostate cancer cells, AR protein levels were examined by immunoblotting of lysates from either CNPY2 or MYLIP knockdown LNCaP cells (Figure 2H). MYLIP knockdown increased AR protein levels whereas CNPY2 knockdown increased MYLIP and reduced AR protein expression levels. These results showed that the E3 ligase MYLIP could ubiquitinate lysine 845 and 847 residues of AR. CNPY2 inhibited the MYLIP-mediated ubiquitination of AR and suppressed AR protein degradation.

### CNPY2 suppresses E3 ligase activity of MYLIP by inhibiting the interaction between MYLIP and UBE2D1

To investigate the mechanism by which CNPY2 inhibited MYLIP function as an E3 ligase, we first examined whether CNPY2 could regulate the auto-ubiquitination activity of MYLIP. MYLIP is a RING finger type E3-ubiquitin ligase [15]. A central function of the RING domain is to form a docking surface for the cognate E2-ubiquitin ligase [14]. A previous report and our results showed that recombinant MYLIP RING protein had auto-ubiquitination activity with E2-ubiquitin ligase UBE2D1 in an *in vitro* ubiquitination system (Figure 3A and 3B) [15]. MYLIP RING-mediated auto-ubiquitination was inhibited by CNPY2 (Figure 3A). To confirm that CNPY2 could decrease the auto-ubiquitination activity of MYLIP, *in vivo* ubiquitination assays were done using 293T cells expressing MYLIP-His, FLAG-CNPY2 and EGFP-ubiquitin. As shown in Figure 3C, accumulation of ubiquitinated MYLIP was expressed in cells treated with MG132. The poly-ubiquitination of MYLIP was inhibited by CNPY2 (Figure 3C). These results suggested that CNPY2 affected an ubiquitination system consisting of E1, E2 and E3-ubiquitin ligases rather than interaction between E3 ligases and its substrate proteins.

We examined whether CNPY2 regulated interaction between E3 ligase MYLIP and E2 ligase UBE2D1. In GST pull-down assays, MYLIP RING bound to UBE2D1, whereas it showed weak interaction with CNPY2 (Figure 3B and 3D). The binding between RING and UBE2D1 was inhibited by CNPY2 (Figure 3D). Consistent with this, interaction between MYLIP and UBE2D1 was inhibited by CNPY2 in 293T cells (Figure 3E). In the cells, CNPY2 showed interaction with UBE2D1 though it scarcely interacted with MYLIP (Figure 3E). These results suggested that CNPY2 regulated the ubiquitination activity of E3 ligase MYLIP through inhibition of physical interaction between MYLIP and an E2 ligase, UBE2D1.

### CNPY2 promotes prostate cancer cell growth through regulation of AR protein level

**Figure 4:**
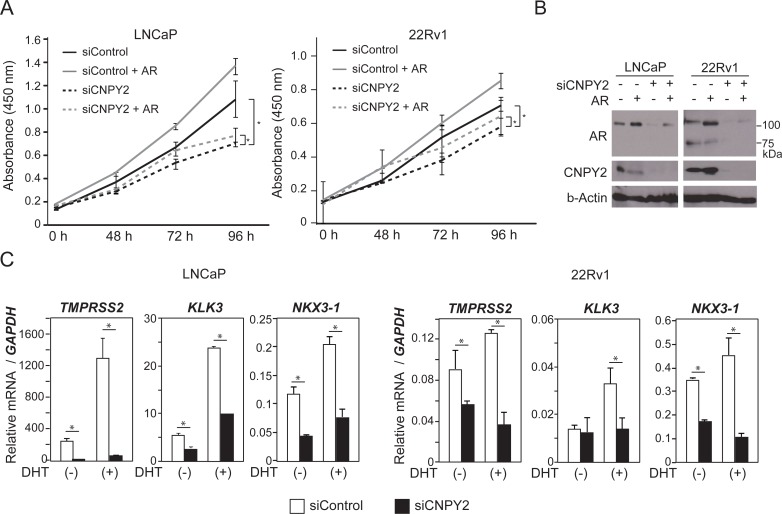
CNPY2 promotes prostate cancer cell growth through regulation of AR expression level (**A**) Cell growth of LNCaP or 22Rv1 cells co-transfected with *CNPY2* siRNA and AR expression plasmids. The cells were cultured in medium with regular FBS. Cell numbers were quantified by determining the absorbance at 450 nm. ^*^P < 0.05. (**B**) Immunoblots using LNCaP or 22Rv1 cells co-transfected with *CNPY2* siRNA and AR expression plasmids. *CNPY2* siRNA or AR expression plasmids were transfected for 4 days or 1 day, respectively. Endogenous AR variants were detected as 75 kDa bands. (**C**) mRNA expression of *TMPRSS2, KLK3* and *NKX3-1* genes in CNPY2 knockdown PCa cells, LNCaP and 22Rv1. Cells were cultured with or without 10^-7^ M dihydrotestosterone (DHT) for 24 h in charcoal stripped 10% FBS-RPMI medium. qPCR was used to measure the expression level of each gene. Each measurement shows the average values of 3 independent measurements, which were normalized to the expression level of *GAPDH* mRNA.^*^P < 0.05.

AR is a ligand-dependent transcription factor that exerts a wide variety of biological actions, including controlling cell growth by altering transcription of target genes [23, 24]. In fact, cell growth of 22Rv1 and LNCaP cells was promoted by overexpression of AR in cultured medium with regular FBS (Figure 4A and 4B). It has been reported that concentration of androgens in regular FBS was sufficient for proliferation of PCa cells [25]. We next examined the function of CNPY2 in cell growth and found that a decrease of CNPY2 expression inhibited growth of 22Rv1 and LNCaP cells (Figure 4A and 4B). This inhibition of cell growth in CNPY2-knockdown cells could be recovered by overexpression of AR (Figure 4A). These results suggested that CNPY2 might promote cell growth through regulation of AR protein levels.

Next, we asked whether CNPY2 could regulate the expression level of AR target genes in prostate cancer. *Transmembrane protease, serin 2* (*TMPRSS2*) [26], *kallikrein related peptide 3 (KLK3/PSA)* [27] and *NK3 homeobox 1 (NKX3-1)* [28] are targets of AR transcriptional control. In 22Rv1 cells, the expression levels of *TMPRSS2* and *KLK3* were relatively low, while *NKX3-1* was highly expressed compared with LNCaP cells (Figure 4C). Transcripts of *TMPRSS2, KLK3* and *NKX3-1* were increased by DHT treatment in both LNCaP and 22Rv1 cells (Figure 4C). CNPY2 knockdown reduced mRNA expression levels of these AR target genes (Figure 4C). In prostate cancer, the 5′-untranslated region of the *TMPRSS2* gene is often translocated to the *v-ets avian erythroblastosis virus E26 oncogene homolog (ERG)* and *ETS variant 1 (ETV1)* gene [26], a member of the ETS gene family that is involved in a wide variety of functions, including cell proliferation [29]. These gene fusions are proposed to be one mechanism by which prostate cancer can progress to androgen independence [30]. Blood tests to measure PSA protein level are used to monitor how well prostate cancer cell growth of patients is suppressed [27]. NKX3-1 is necessary to prostate development [31], and promotes cell proliferation of prostate cancer cells [32]. From the above results, CNPY2 increased AR protein levels and promoted AR transactivation of the AR target genes, which might promote cell growth of prostate cancer.

### CNPY2 expression positively correlated with expression of AR and AR target genes in prostate cancer patients

**Figure 5:**
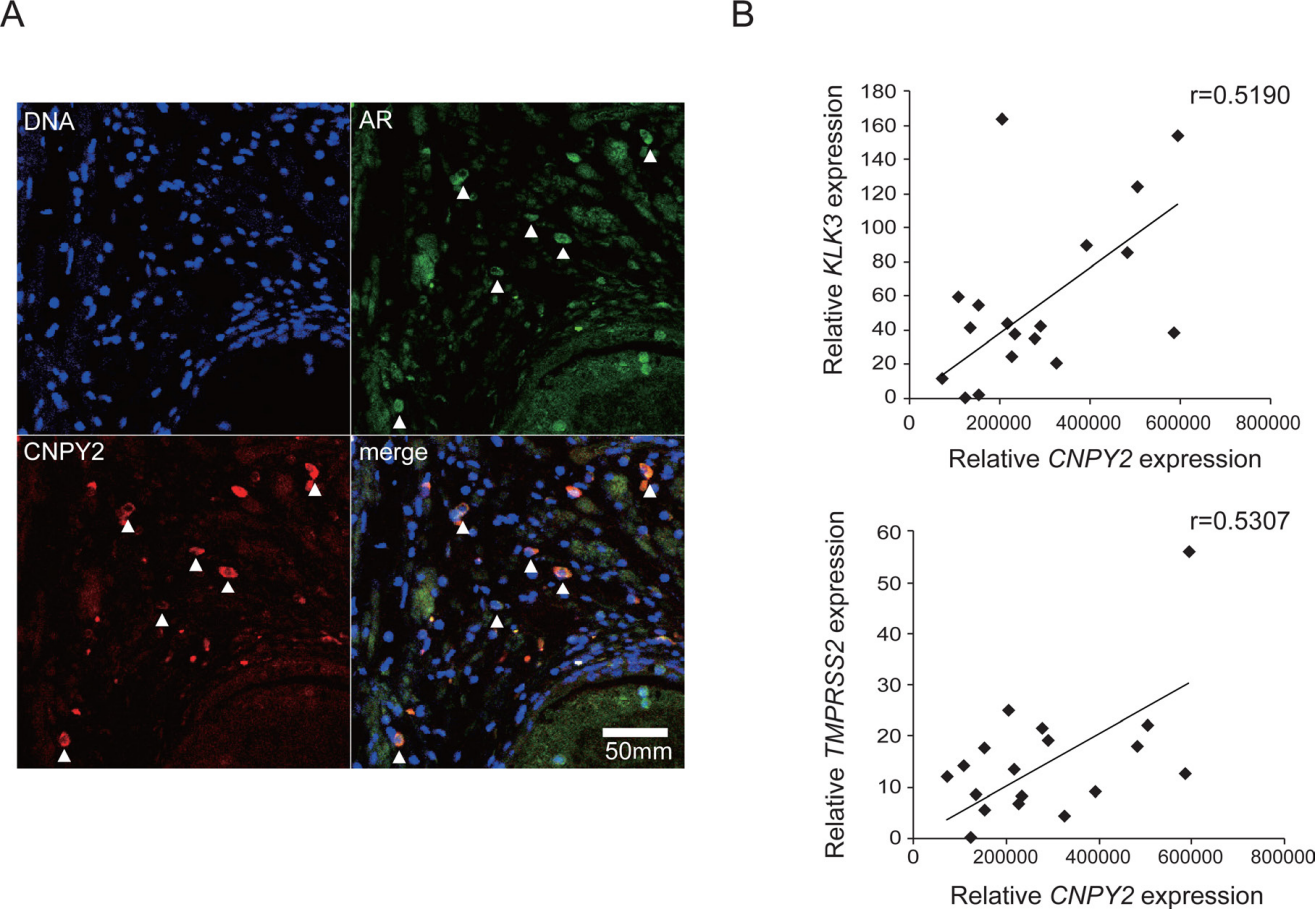
CNPY2 expression correlated with AR and AR target genes expression in prostate cancer patients (**A**) Immunostaining using human prostate cancer tissues. DNA was stained with DAPI (blue). AR (green) and CNPY2 (red) were detected by immunostaining to demonstrate co-localization in some cells. (**B**) *CNPY2*, *KLK3* and *TMPRSS2* mRNA expression in prostate cancer tissues (*n* = 18). mRNA expression levels were quantified by qPCR. Each measurement was normalized to *GAPDH* mRNA levels.

To investigate whether there was a correlation between CNPY2 protein expression and AR protein expression in prostate cancer, we examined CNPY2 and AR in primary prostate cancer tissues (Figure 5A). Using specific antibodies against CNPY2 and AR, immunohistochemistry showed that, as with the cell studies, CNPY2 and AR were co-localized in the prostate cancer tissues of patients. To assess the correlation between CNPY2 and AR expression levels more precisely, mRNA expression levels of *CNPY2* and *AR* target genes (*KLK3* or *TMPRSS2*) were quantified by qPCR in tissues from prostate cancer patients (*n* = 18; Supplementary Table 1). The result suggested the presence of a positive correlation between *CNPY2* and *AR* target genes expression levels (KLK3: *r* = 0.5190, TMPRSS2: *r* = 0.5307) in these prostate cancer patient samples (Figure 5B), indicating that CNPY2 expression was positively correlated with AR expression and AR transcriptional activation in prostate cancer patients.

## DISCUSSION

In this study, we showed that MYLIP is a novel E3 ubiquitin ligase that recognizes the AR as its target protein in prostate cancer cells. Furthermore, we suggest that CNPY2 increased AR protein levels by preventing MYLIP-mediated AR ubiquitination, promoting gene expression of AR target genes such as *TMPRSS2, KLK3* and *NKX3-1*. CNPY2 repressed ubiquitination activity of MYLIP by inhibiting the association with MYLIP and UBE2D1, an E2 ubiquitin ligase. These results suggested that CNPY2 may promote PC progression by increasing AR protein levels.

A previous report showed that MYLIP RING domain-UBE2D interaction was important for protein degradation of its target protein, LDLR [14]. Though CNPY2 was reported to inhibit the function of MYLIP as an E3 ubiquitin ligase [11], the mechanism remains unclear. We suggest that CNPY2 inhibits the physical interaction between MYLIP and UBE2D1 and degradation of the target proteins. Various inhibitors of the ubiquitin proteasome system have been developed, and some of them are used as drugs for cancer treatment. E3 ligases are particularly responsible for substrate specificity, therefore inhibition of an E3 is effective for control expression level of the specific protein. Structural analysis of CNPY2 interaction with UBE2D1 is required to improve our understanding of how CNPY2 inhibits MYLIP-UBE2D1 interaction, which may lead to development of new targeted therapy for cancer.

We previously identified CNPY2 as a PC cell growth promoter by genetic screening of a prostate cancer model in *Drosophila* [10]. The fly homologue of human CNPY2, *seele (sel)*, promoted cell growth in homologous organs of human prostate (accessory gland) [10], though AR was not expressed in the fly. This fact and the results showing that cell growth suppression by human CNPY2 knockdown could not be fully rescued by AR overexpression (Figure 4A) indicate that CNPY2 may promote PC cell growth through additional known mechanisms other than AR protein stabilization. Investigations into the function of seele in cell growth in *Drosophila* accessory glands may reveal other functions of CNPY2 in addition to AR stabilization.

The mechanism for regulation of CNPY2 expression remains unclear, but some evidence suggests that hypoxia may play a role. Interestingly, the *CNPY2* promoter region carries a hypoxia responsive element (HRE). Androgen deprivation by castration causes hypoxia due to a reduction of blood flow in the prostate and increased expression of HIF-1α [33]. A previous report suggested that hypoxic stimulation induces HIF-1α expression, which in turn activates the *CNPY2* promoter and promotes *CNPY2* expression in human smooth muscle cells [34]. It is conceivable that hypoxia caused by castration may lead to hyper-expression of *CNPY2* through the activation of the HRE in the *CNPY2* promoter region in prostate cancer cells. Future studies could examine fluctuations in *CNPY2* expression induced by castration in model animals such as rats.

Given the critical role of AR in castration resistant-prostate cancer (CRPC) progression, promotion of AR degradation may be a promising target for CRPC patient therapy. ASC-J9, an AR degradation enhancer, suppresses CRPC cell growth through degradation of full-length and splice variant ARs [35]. MDM2 and E6-AP are E3 ligases that interact with the AR protein [36, 37]. Moreover, emodin, a natural compound present in Japanese knotweed and rhubarb, is known to induce AR protein degradation by increasing the association between AR and Mdm2, [38] and also to suppress prostate cancer growth in both *in vitro* and *in vivo* models. The expression of E6-AP protein in invasive prostate cancer was lower than that of adjacent normal tissue [39], whereas E6-AP overexpression in stable cell lines resulted in decrease of AR protein level [37]. In conclusion, results from our study suggested that the MYLIP E3 ligase could be a new therapeutic target for CRPC based on its ability to recognize AR and induce AR protein degradation.

## MATERIALS AND METHODS

### Plasmids, siRNAs, and antibodies

For expression in cultured cells, EGFP tag-fused ubiquitin, FLAG-CNPY2, FLAG-MYLIP, MYLIP-His, FLAG-UBE2D1, UBE2D1-His and deletion mutants of FLAG-ARs (full length, 1-920 a.a.; AF-2, 555-920 a.a.; AF-1, 1-673 a.a.) were inserted into the pcDNA3 vector (Invitrogen, Carlsbad, CA, USA). To produce recombinant proteins in *E.coli*, CNPY2, UBE2D1 and the RING domain of MYLIP (344-445 a.a.) were inserted into pET29a (+) vector (Merck Millipore, DA, Germany) or the pGEX4T1 vector (GE Healthcare, Little Chalfont, England). siRNAs for *CNPY2* (CNPY2HSS115810 and 115811) and *MYLIP* (MYLIPHSS120911 and 120912) were purchased from Invitrogen. Data in this report was shown as results using siCNPY2 (115810) and siMYLIP (120911), which had been more efficient at knockdown or cell growth than siCNPY2 (115811) in 22Rv1 cells. A nonspecific control siRNA pool (siControl) was purchased from Dharmacon (D-001206-13-20). The following antibodies were used: AR (N-20, C-19 or 441, Santa Cruz Biotechnology, Santa Cruz, CA, USA), CNPY2 (ab181215, Abcam, Cambridge, MA, USA), MYLIP (ab74562, Abcam), β-actin (A5441, Sigma, St. Louis, MO, USA), Ub (F-11, Santa Cruz Biotechnology), FLAG (F7425, Sigma), His (D291-3S, MBL, Nagoya, Japan), GST (B-14, Santa Cruz Biotechnology) and GFP (598, MBL).

### Cell culture and transfection

LNCaP and 293T cells were provided by the RIKEN BRC through the National Bio-Resource Project of the MEXT, Japan. 22Rv1 cells were purchased from ATCC. LNCaP and 22Rv1 cells were cultured in RPMI1640 (Nacalai Tesque, Kyoto, Japan) with 10% fetal bovine serum (FBS) at 37° C under 5% CO_2_. When cells were treated with DHT (5α-androstan-17β-ol-3-one; Sigma), charcoal stripped FBS was used for the cell culture. 293T cells were cultured in Dulbecco’s modified Eagle’s medium (DMEM, Nacalai Tesque) with 10% FBS at 37° C under 5% CO_2_. MG132 (Merck Millipore) was added to the culture medium at a concentration of 10 µM 4 or 5 h before cell extraction. To knockdown cellular expression, siRNA transfection was performed using Lipofectamine RNAiMAX (Invitrogen) with antibiotic-free medium for more than 3 days. Transfection of cDNAs into 293T cells was performed using Lipofectamine 2000 (Invitrogen) for 24 h.

### Cell growth assay

22Rv1 or LNCaP cells were transfected with siRNAs using Lipofectamine RNAiMAX for 3 days. The transfected cells were transferred to 96 well plates at 5,000 cells per well. Living cells were then counted using the Kit-8 cell counting method (Dojindo, Kumamoto, Japan).

### RNA isolation and qPCR

Total RNA was isolated using ISOGEN reagent (Wako). Reverse transcription (RT) was performed using PrimeScript RT Master Mix (Takara, Shiga, Japan) according to the manufacturer’s instructions. cDNAs were quantified by real-time PCR using SYBR qPCR mix (Toyobo, Osaka, Japan) and a Thermal Cycler TP800 (Takara). RT primers were as follows: *AR* (s) 5′-ATGGTGAGCAGAGTGCCCTA-3′, (as) 5′-TCTGGGGTGGAAAGTAATAGTCAA-3′; *CNPY2* (s) 5′- GACCATGCCCTGCACATATC-3′, (as) 5′- TAAAAGGCATTGCCACCATT-3′; *GAPDH* (s) 5′-GCACCGTCAAGGCTGAGAAC-3′, (as) 5′-TGGTGAAGACGCCAGTGGA-3′; *KLK3* (s) 5′- TCTGCGGCGGTGTTCTG-3’, (as) 5′- GCCGACCCAGCAAGATCA-3′; *TMPRSS2* (s) 5′- GGACAGTGTGCACCTCAAAGAC-3′, (as) 5′- TCCCACGAGGAAGGTCCC-3′; *NKX3-1* (s) 5′- CCCAGTCCACTGAGCAAGCA-3′, (as) 5′- GGGACCCATTATAGGCAATAAACAC-3′.

### Western blotting

Whole cell lysates were extracted with lysis buffer (10 mM Tris-HCl [pH 7.8], 1% NP-40, 0.15 M NaCl, 1 mM EDTA). Western blotting was then performed using standard methods [40].

### Immunoprecipitation

To determine the interaction between AR and MYLIP, LNCaP or 293T cells were transfected with expression plasmids for 24 h. Whole cell extracts of the cells were prepared as previously described [40]. For immunoprecipitation, cellular lysates were incubated with anti-FLAG M2 affinity gel (Sigma) or Dynabeads His-Tag Isolation and Pulldown (ThermoFisher Scientific, Waltham, MA, USA) at 4° C for 2 h. Proteins bound to anti-FLAG-beads or anti-His beads were resolved by SDS-PAGE and detected by immunoblotting.

### *In vivo* ubiquitination assay

To detect ubiquitination of AR (AF-2), 293T cells were transfected with FLAG-ARs (AF-2, K845R or K847R), MYLIP-His and EGFP-Ubiquitin expression plasmids for 24 h. The transfected cells were incubated with 10 µM MG132 for 5 h. Whole cell extracts of 293T cells were incubated with anti-FLAG M2 affinity gel at 4° C for 2 h. The resulting immunoprecipitates were then subjected to Western blotting. To detect ubiquitination of AR (full length), 293T cells were transfected with AR (full length), FLAG-MYLIP, EGFP-Ubiquitin and FLAG-CNPY2 expression plasmids for 24 h and incubated with 10 µM MG132 for 5 h. Whole cell extracts of the 293T cells were incubated with anti-AR (N-20) at 4° C for 16 h before incubation with Dynabeads Protein G (Life Technology, Oslo, Norway) for 1 h at 4° C. To detect auto-ubiquitination of MYLIP, 293T cells were transfected with MYLIP-His, EGFP-Ubiquitin and FLAG-CNPY2 expression plasmids for 24 h and incubated with 10 µM MG132 for 5 h. Immunoprecipitations were performed with Dynabeads His-Tag Isolation and Pulldown at 4° C for 2 h using cell lysates of 293T cells.

### *In vitro* ubiquitination assay

Recombinant GST-fused CNPY2 and MYLIP partial fragment (RING) were produced in BL21 (DE3) bacterial cells (BioDynamics Laboratory Inc., Tokyo, Japan), purified with Glutathione Sepharose beads (GE Healthcare) and eluted with 40 mM reduced glutathione (pH 8.0). His-fused AR (AF-2) was produced in BL21 (DE3) bacterial cells, and purified with Dynabeads His-Tag Isolation and Pulldown. Transiently expressed FLAG-fused MYLIP in 293 T cells was purified with anti-FLAG M2 affinity gel (Sigma) and eluted with FLAG peptide (Sigma).

*In vitro* ubiquitination assays were performed as described with modifications [41, 15]. 1.5 nmol recombinant ubiquitin (U-100H, Boston Biochem, Cambridge, MA, USA), 33 ng of recombinant UBE1 (E-305, Boston Biochem), 34 ng of recombinant UbcH5a/UBE2D1 (E2-616, Boston Biochem), 500 ng of FLAG-MYLIP, 500 ng of recombinant AR (AF-2)-His, 500 ng of recombinant GST-CNPY2 and 100 nmol of ATP (Roche, Basel, Switzerland). Reagents were incubated at 37° C for 2 h in ubiquitination buffer [25 mM Tris-HCl (pH8.0), 100 mM NaCl, 5 mM MgCll_2_, 1 mM DTT]. In experiments to detect auto-ubiquitination of MYLIP, 300 ng of GST-RING and 2 μg of GST-CNPY2 were used for the reaction. Reactions were stopped by addition of SDS-PAGE loading buffer and subjected to immunoblotting.

### GST pull-down assay

Recombinant GST-RING (8 μg) plus Glutathione Sepharose beads were incubated with recombinant UBE2D-His (5 μg) or immunoprecipitated FLAG-CNPY2 (5 or 15 μg) at 4° C for 30 min in binding buffer [50 mM Tris-HCl (pH 7.5), 150 mM NaCl, 5 mM EDTA (pH 7.9), 0.5% NP40, 1 mM PMSF, 1 μg/mL aprotinin], washed with binding buffer and subjected to immunoblotting.

### Patient tumor samples

Human prostate tumor samples were obtained from University Hospital, Kyoto Prefectural University of Medicine, in accordance with protocols approved by the Hospital’s Institutional Ethical Committee. Written informed consent was obtained from each patient. Tumor samples were taken from freshly isolated surgical resections or needle biopsy.

### Immunohistochemistry

Prostate cancer patient samples were embedded in paraffin. Immunostaining and analyses were carried out as previously described [5, 40]. Serial sections of prostate cancer tissues were deparaffinized, rehydrated, and washed in distilled water before incubation in Trilogy solution (Cell Marque, CA, USA) for 40 min using a steam cooker. After blocking in 5% skim milk in PBST for 1 h, sections were incubated with primary antibodies (α-AR 441 and α-CNPY2) in 0.5% skim milk/PBST overnight at 4° C. Sections were then incubated with fluorescent antibodies (Jackson ImmunoResearch, PA, USA) for 1 h at 25° C and mounted on slides using VECTASHIELD mounting medium with DAPI (Vector Laboratories, CA, USA). Immunofluorescent staining was visualized using a Zeiss 510 laser confocal microscope.

### Statistical analysis

Statistical analyses were carried out by *t*-test as appropriate. All data are reported as means ± SD. A *P*-value of < 0.05 was considered significant.

## SUPPLEMENTARY MATERIALS TABLE


