# Supplementary material for: CNPY2 inhibits MYLIP-mediated AR protein degradation in prostate cancer cells
Source: Oncotarget. 2018 Apr 3;9(25):17645–55. doi: 10.18632/oncotarget.24824 (PMC5915145; doi:10.18632/oncotarget.24824)
Supplement: Supplementary file 1 [file oncotarget-09-17645-s001.pdf]

CNPY2 inhibits MYLIP-mediated AR protein degradation in prostate cancer cells

SUPPLEMENTARY MATERIALS

Supplementary Table 1: Clinical features of prostate tumors

No.	Age	GS	Tissue	Types of biopsy procedures	Histology	Stage
1	67	5+4	prostate	prostate needle biopsy	adenocarcinoma	cT4N1M1
2	64	4+5	prostate	prostate needle biopsy	adenocarcinoma	cT4N1M1
3	73	3+4	prostate	prostate needle biopsy	adenocarcinoma	cT3bN1M0
4	86	5+4	prostate	prostate needle biopsy	adenocarcinoma	cT3aN0M1
5	71	3+5	prostate	total prostatectomy	adenocarcinoma	pT2bN0M0
6	76	4+4	prostate	prostate needle biopsy	adenocarcinoma	cT4N0M1
7	87	5+4	prostate	prostate needle biopsy	adenocarcinoma	cT3aN0M0
8	83	4+5	prostate	prostate needle biopsy	adenocarcinoma	cT3bN0M1
9	75	5+4	prostate	prostate needle biopsy	adenocarcinoma	cT4N0M1
10	76	5+5	prostate	prostate needle biopsy	adenocarcinoma	cT3aN2M1
11	84	4+4	prostate	prostate needle biopsy	adenocarcinoma	cT4N1M1
12	76	5+4	prostate	prostate needle biopsy	adenocarcinoma	cT4N2M1
13	55	3+4	prostate	prostate needle biopsy	adenocarcinoma	cT4N0M1
14	63	4+5	prostate	prostate needle biopsy	adenocarcinoma	cT4N2M1
15	69	3+4	prostate	total prostatectomy	adenocarcinoma	pT2bN0M0
16	70	4+4	prostate	prostate needle biopsy	adenocarcinoma	cT2bN0M0
17	87	5+4	prostate	prostate needle biopsy	adenocarcinoma	cT4N0M1
18	78	5+4	prostate	prostate needle biopsy	adenocarcinoma	cT3aN0M1

GS: Gleason score.
